# Study on Protection Mechanism of 30CrMnMo-UHMWPE Composite Armor

**DOI:** 10.3390/ma10040405

**Published:** 2017-04-12

**Authors:** Yu Zhou, Guoju Li, Qunbo Fan, Yangwei Wang, Haiyang Zheng, Lin Tan, Xuan Xu

**Affiliations:** 1 School of Materials Science and Engineering, Beijing Institute of Technology, Beijing 100081, China; dahuabit@163.com (Y.Z.); psq112358@msn.com (G.L.); wangyangwei@bit.edu.cn (Y.W.); 2 National Key Laboratory of Science and Technology on Materials under Shock and Impact, Beijing 100081, China; 3 Beijing Winyarn High Performance Fiber Company Limited, Beijing 101407, China; polpol6720@sina.com (H.Z.); snova@139.com (L.T.); biblias@163.com (X.X.)

**Keywords:** 30CrMnMo-UHMWPE, finite element analysis, ballistic performance, protection mechanism

## Abstract

The penetration of a 30CrMnMo ultra-high molecular weight polyethylene armor by a high-speed fragment was investigated via experiments and simulations. Analysis of the projectile revealed that the nose (of the projectile) is in the non-equilibrium state at the initial stage of penetration, and the low-speed regions undergo plastic deformation. Subsequently, the nose-tail velocities of the projectile were virtually identical and fluctuated together. In addition, the effective combination of the steel plate and polyethylene (PE) laminate resulted in energy absorption by the PE just before the projectile nose impacts the laminate. This early absorption plays a positive role in the ballistic performance of the composite armor. Further analysis of the internal energy and mass loss revealed that the PE laminate absorbs energy via the continuous and stable failure of PE fibers during the initial stages of penetration, and absorbs energy via deformation until complete penetration occurs. The energy absorbed by the laminate accounts for 68% of the total energy absorption, indicating that the laminate plays a major role in energy absorption during the penetration process.

## 1. Introduction

With the rapid development of modern weapons, the development of new types of ballistic-resistant light-weight high-performance armor structures has become increasingly important in recent years. High-performance armor must exhibit excellent resistance to penetration, impact, and caving. However, a single-structure armor typically satisfies only some of these requirements. Nevertheless, the ballistic performance can be realized and the areal density of the armor can be reduced by developing composite-structure armors that fully exploit the properties of each material.

Composite-structure armor usually consists of a front plate that can prevent initial penetration of the projectile, and a back plate that can absorb the energy without fracture and caving; energy absorption is prevented owing to the high toughness of the back plate at the end of penetration. In general, the development of this type of armor has focused on ceramics and fiber composite structures [1,2,3,4,5,6,7]. Greenhalgh et al. observed on Dyneema^®^ laminates which had been exposed to ballistic impact, and the influence of processing conditions and target response on the damage processes had been gleaned [5]. Sherman investigated the quasi-static and the dynamic damage mechanisms in alumina tiles backed by Spectra^®^ support plates under a variety of boundary conditions [6]. By using explicit finite element analysis, Krishnan et al. built the models in which excellent predictions of damage were made and a high-performance armor package was designed and optimized [7]. Low-density high-hardness ceramics can abrade, blunt, and fracture the projectile, leading to a decrease in the penetration performance of the projectile [6]. However, ceramic plates are brittle and have low tensile strength and are, therefore, typically used in combination with fiber back plates to improve the integrated protective properties of the composite armor [8,9,10].

In addition, metal and fiber composite structure armors have received increasing attention for use in practical applications. Masta et al. compared the protective performance of in-house-developed Al/ultra-high molecular weight polyethylene (UHMWPE) and Al/Al_2_O_3_-UHMWPE composite armor. They found that the deforming Al/Al_2_O_3_ panel preaccelerated the laminate before the arrival of fragment and distributing the impact momentum might suppress local laminate failure, resulting in a higher impact resistance of Al/Al_2_O_3_-UHMWPE composite armor [11]. Similarly, Haro investigated the ballistic impact behavior of hybrid composite laminates fabricated from aluminum and Kevlar^®^. Deformation analysis and penetration behavior of the targets were studied in different stages, and mechanisms of failure were investigated using SEM examination of the perforations [12]. However, the protection mechanism of this composite structure must be elucidated. New high-strength high-toughness alloy steel, suitable for use in the armor front plate, has been developed in recent years [13,14]. In fact, an understanding of the protective performance of metal and fiber composite armor is essential for use of these armors in vehicles and ships mainly composed of metal structures.

In this work, the ballistic performance of metal-fiber composite armor is investigated with the aim of revealing its protective mechanism. A medium carbon low alloy steel, 30CrMnMo steel, is selected owing to its excellent balance of strength and toughness, and widespread use in the military industry [15]. UHMWPE, a third-generation fiber (after glass fiber and aramid fiber) for projectile resistance, has high strength, high modulus, and low density [16]. This fiber offers excellent protection against small fragment and bullets [17,18] and is, hence, widely used in various types of individual protection products. Therefore, in this work, a 30CrMnMo-UHMWPE composite armor is investigated with the aim of revealing the protective mechanism of this new armor structure. Under the impact of a high-speed fragment, the dynamic response of the projectile and the energy absorption of each region in the composite armor is determined via experiments and finite element method (FEM) simulations. The matching between the metal and PE laminate during deformation is discussed, and the protection mechanism of the metal-fiber composite structure armor is identified.

## 2. Ballistic Test and Modeling

### 2.1. Experimental Details and Geometrical Models

**Figure 1:**
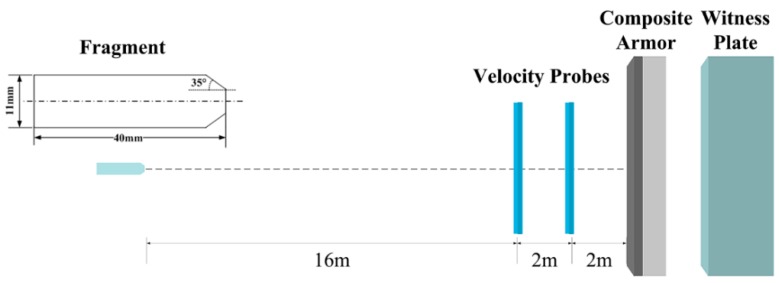
Schematic of the experimental setup.

Fragment simulating projectiles are the reference penetrators which are used to simulate artillery shell fragments [19,20]. The shape is non-axisymmetric, and the dimensions and material properties fulfill the requirements of Military Specification MIL-P-46593A [21]. In this work, a Φ11 mm × 40 mm 1045-steel fragment with a wedge-shaped nose, is used in the experiments. With an area of 300 × 300 mm^2^, the composite-structure armor consists of an 8 mm-thick 30CrMnMo plate and a 21 mm-thick UHMWPE laminate. A 60 mm-thick aluminum alloy plate, placed 42-mm behind the laminate, serves as a witness plate. Boundaries of the composite armor are fully-constrained, and the velocity of normal impact is 1798 m/s. A schematic of the experimental setup is shown in Figure 1.

**Figure 2:**
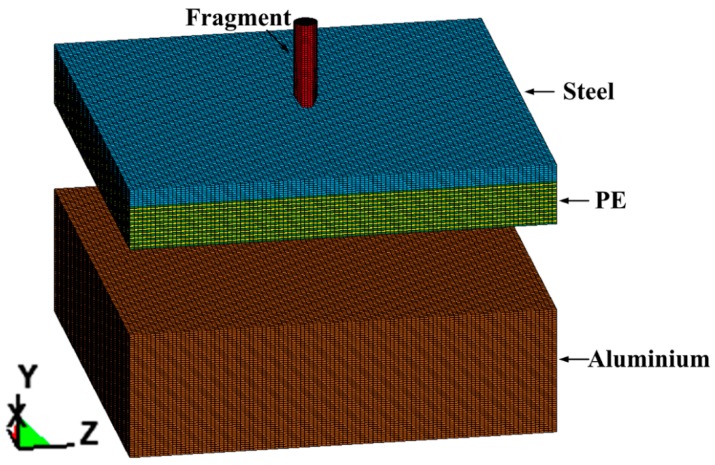
Initial state of the 3D FEM (finite element method) model.

The progress of projectile-target interaction is simulated using the non-linear dynamics finite element software LS-Dyna. The three-dimensional (3D) model is composed of three parts, namely the: fragment, steel-PE composite armor, and witness plate; the initial state of the model is shown in Figure 2.

### 2.2. Material Models and Parameters

The fragment, steel plate, and aluminum alloy plate are described by the Johnson–Cook constitutive model (*MAT_JOHNSON_COOK). This model takes the strain-hardening effect and the strain-rate effect into consideration, and is widely used for the simulation of severe-plastic-deformation behavior of metals under high-strain rate conditions (including metal explosive forming and ballistic penetration and impact). The constitutive equation of this model is given as follows:

(1) $$\sigma_{y}=\left(A+{B\varepsilon }^{p^{n}}\right)\left(1+{\mathrm{Cln}\varepsilon }^{*}\right)\left(1-T^{*m}\right)$$

where, σ_y_: dynamic yield stress, ε^p^: effective plastic strain, ε^*^: normalized effective strain-rate, T*: homologous temperature ($T^{*}=\frac{T-T_{r}}{T_{m}-T_{r}}$; T, T_r_ and T_m_ denote the current temperature, room temperature, and melting point, respectively). Furthermore, the parameters A, B, C, n and m represent the static yield stress, hardening parameter, strain rate parameter, hardening index, and temperature index, respectively. For 1045-steel, 30CrMnMo and aluminum, the strain at fracture (ε^f^) in Johnson-Cook constitutive model is given by Formula (2) with material parameters D_1_ to D_5_:

(2) $$\varepsilon^{f}=\left(D_{1}+D_{2}{\mathrm{expD}}_{3}\sigma^{*}\right)\left(1+D_{4}\ln\varepsilon \right)\left(1+D_{5}T^{*}\right)$$

For a given element, the damage parameter $D=\sum \frac{∆\varepsilon^{p}}{\varepsilon^{f}}$ accumulates with the increase of material deformation. Fracture occurs when D reaches the value of 1, which leads to the element failure and deletion. The material parameters used in Johnson-Cook model listed in Table 1 are obtained by using Hopkinson bar test [22].

PE laminates are fabricated via orthogonal hot-pressing of PE fibers and, in this work, are described by the orthotropic elastic material model (*MAT_ORTHOTROPIC_ELASTIC) [23,24]. Each point in this model is intersected by three pairs of perpendicular planes. The direction perpendicular to the symmetry plane is referred to as the elastic principal direction. The axis parallel to the elastic principal direction is referred to as the elastic spindle axis or material axis, and is generally denoted as 1, 2 and 3. In the material-axis coordinate system of this model, the relationship between the strain and stress components is given as follows:

(3) $${\left\{\varepsilon \right\}}_{m}={\left\{C\right\}}_{m}\times {\left\{\sigma \right\}}_{m}$$

where, ${\left\{\varepsilon \right\}}_{m}=\left\{\varepsilon_{11}\varepsilon_{22}\varepsilon_{33}\gamma_{12}\gamma_{23}\gamma_{31}\right\}$ and ${\left\{\sigma \right\}}_{m}=\left\{\sigma_{11}\sigma_{22}\sigma_{33}\tau_{12}\tau_{23}\tau_{31}\right\}$. ${\left\{C\right\}}_{m}$ is the stiffness matrix of the material, which is given as:

(4) $${\left\{C\right\}}_{m}=\left[\begin{array}{cccccc} \frac{1}{E_{1}} & -\frac{\mu_{21}}{E_{2}} & -\frac{\mu_{31}}{E_{3}} & 0 & 0 & 0\\ -\frac{\mu_{12}}{E_{1}} & \frac{1}{E_{2}} & -\frac{\mu_{32}}{E_{3}} & 0 & 0 & 0\\ -\frac{\mu_{13}}{E_{1}} & -\frac{\mu_{23}}{E_{2}} & \frac{1}{E_{3}} & 0 & 0 & 0\\ 0 & 0 & 0 & \frac{1}{G_{12}} & 0 & 0\\ 0 & 0 & 0 & 0 & \frac{1}{G_{23}} & 0\\ 0 & 0 & 0 & 0 & 0 & \frac{1}{G_{31}} \end{array}\right]$$

The material parameters used in the model are listed in Table 1 (ρ: density and PR: Poisson’s ratio). A, B and C correspond to the (three material axes 1, 2 and 3, respectively. Therefore, EA, EB, and EC correspond to the Young’s modulus along these respective axes. Similarly, GAB and PRBA represent the shear modulus and Poisson’s ratio along the respective direction. In orthotropic elastic model, a maximum principal stress failure criterion is used in the modeling [25]. When the maximum principal stress reaches the peak value of 2.3 GPa, the corresponding elements become failed and deleted automatically from the mesh. The material parameters of UHMWPE for orthotropic elastic model are obtained by using planar plate impact test [26].

### 2.3. Other Details

The calculation models use 8-node hexahedral elements. The fragment and the armor plates are meshed using a refinement mapping method [27]. The 3D element formulation is constant stress solid element, and reduced integration is used. The hourglass control type is 4 in LS-DYNA, which means the hourglass is in the form of Flanagan-Belytschko stiffness [28,29]. Each layer of FE mesh represents a number of PE layers [7], and the contact type between the armors is enforced via *CONTACT_AUTOMATIC_SURFACE_TO_SURFACE. The keyword *CONTACT_AUTOMATIC_SURFACE_TO_SURFACE_TIEBREAK is used to describe the bonding between the PE fiber layers inside the PE laminate; the normal failure stress is 1.0 GPa by using T-peel test [30] and the shear failure stress is measured to be 0.1 GPa by using single lap-joint shear test [31,32].

## 3. Results and Discussion

### 3.1. Reliability Verification

**Figure 3:**
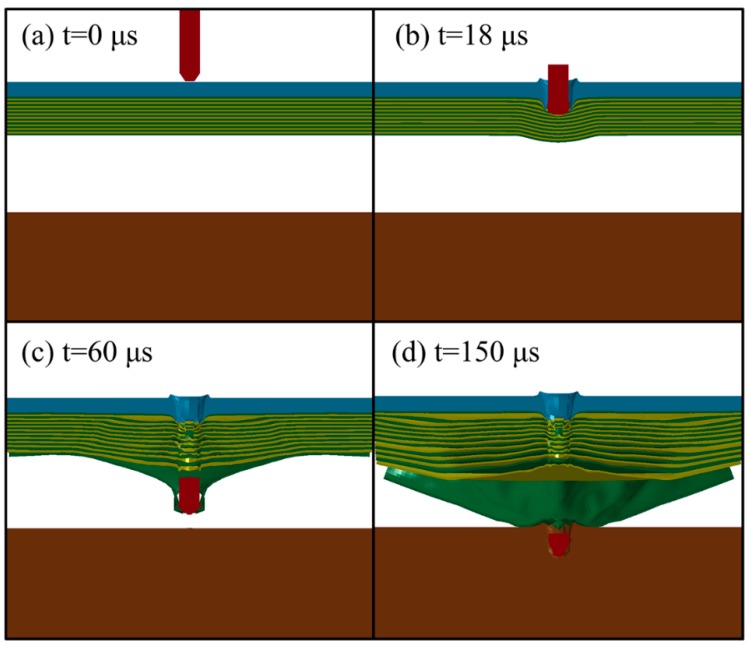
Simulation results showing penetration at different times (**a**) 0 μs (**b**) 18 μs (**c**) 60 μs (**d**) 150 μs.

Figure 3 shows the finite element simulation results of the fragment penetrating the composite armor at various times. The initial state at 0 μs, and the steel and PE laminates penetrated at 18 μs and 60 μs are shown in Figure 3a–c, respectively. At 150 μs, the fragment becomes stuck in the witness plate (see Figure 3d).

**Figure 4:**
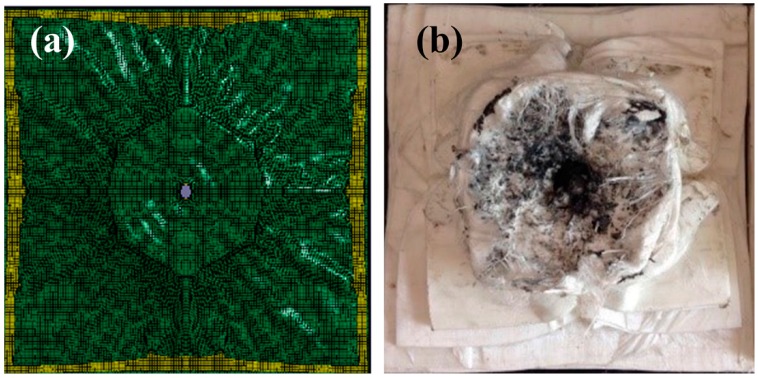
Backfaces of the PE laminate after the (**a**) FEM simulation; and (**b**) ballistic test.

The back morphology of the PE laminate after the ballistic test is compared with the FEM-simulated morphology (see Figure 4). Consistent with the test result, the simulation result (Figure 4a) indicates that several back layers of the laminate coalesce to form a large back bulge during fragment penetration (Figure 4b).

The penetration depths in the witness plate and the residual mass of fragments are obtained and compared between the experiment and the numerical simulation, as shown in Table 2. It can be seen that the numerical simulation result agrees well with the experimental result. Considering the initial velocities of fragment for the simulation and the test are the same of 1798 m/s, it can be deduced indirectly that they have the similar residual velocities and residual kinetic energy.

### 3.2. Penetration Mechanism of the Projectile

**Figure 5:**
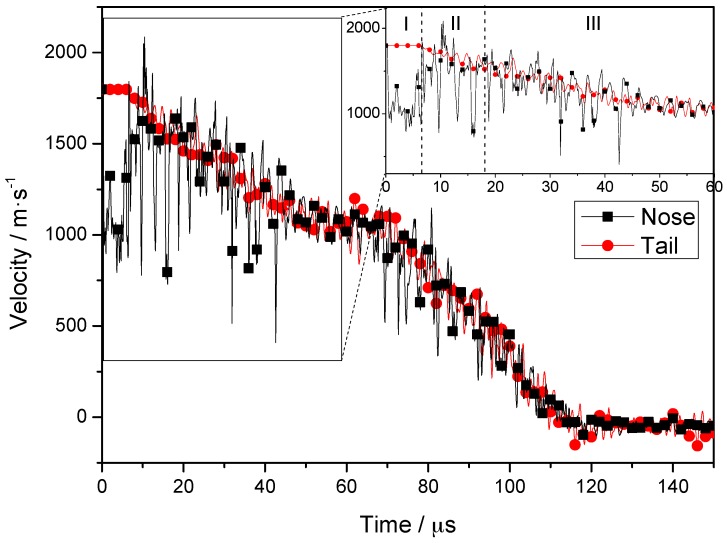
Node velocities at nose and tail during penetration.

The velocities associated with fragment penetration of the composite armor are plotted as a velocity-time curve (Figure 5); the velocities of nodes on the central axis from the nose to the tail of the projectile are considered. The inset shows a magnified view of the velocities associated with times of 0–60 μs. Based on Figure 5, the penetration process can be divided into three stages. In stage I (0–6.5 μs), the velocity of the tail remains constant, whereas the velocity of the nose decreases initially and then increases to almost the same value as that of the tail. In stage II (6.5–18 μs), the steel plate is completely penetrated by the projectile. The velocities of the nose and tail of the projectile decrease in the same manner, although the fluctuation in the velocity of the nose is more significant. In stage III (18–60 μs), the projectile penetrates the PE laminate, and the velocity of the nose and tail are almost the same. The whole penetration process can be divided into three stages by 6.5 μs and 18 μs, and 12 μs is the middle of stage II. As the timestep of simulation result is 2 μs, so we choose 6, 12 and 18 μs as typical time for further analysis.

In stage I, the stress generated when the nose reaches the target is far greater than the yield stress of the projectile material and, hence, plastic deformation and element failure occurs in the nose. Simultaneously, the velocity of the nose decreases from an initial value of 1798 m/s to ~1000 m/s. The velocity increases to 1700 m/s as the high-speed tail moves forward, and the velocities of the nose and tail become basically the same. An elastic wave speed ($v=\sqrt{E/\rho }$) of 5075 m/s is obtained for the projectile. Considering that the initial length of the projectile is 40 mm, a maximum time of 7.8 μs is required for transmission of the wave from the nose to the tail. The simulation results (see Figure 5) show that the velocity of the tail remains constant at first, and then decreases after 6.5 μs. This indicates that a time of 6.5 μs is required for transmission of the stress wave. The slight differences between the simulation results and the theoretical predictions may have resulted from the wedge structure in the nose and erosion-induced shortening of the projectile [33].

**Figure 6:**
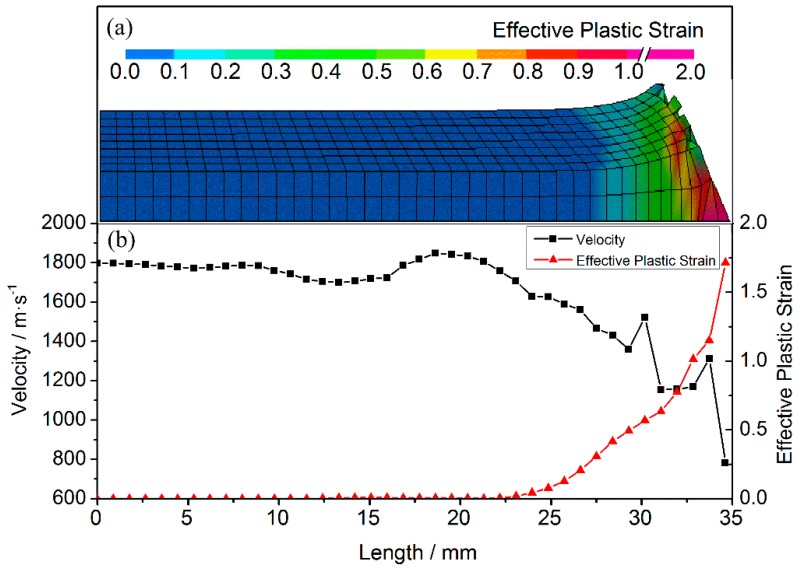
Simulation results at 6 μs. (**a**) Contours of equivalent plastic strain; and (**b**) node velocities and the corresponding element equivalent plastic strains.

The node velocities and the element equivalent plastic strain in various parts (see Figure 6) are plotted to reveal the relationship between the velocity and the deformation in different parts of the projectile. These velocities at a typical time of 6 μs are plotted along a local coordinate, which describes the distance from the tail to the nose along the medial axis of the projectile. Owing to erosion during penetration, the length of the projectile is reduced to 35 mm. The plastic strain is zero for lengths of 0–22 mm, indicating that only elastic deformation occurs and, hence, the high initial values (~1798 m/s) of the node velocities are maintained. Owing to the elastic waves, however, the velocity fluctuates significantly near the nose. Furthermore, at projectile lengths of 22–35 mm, the equivalent plastic strain values of the projectile elements increase gradually from 0.006, and the speed of the corresponding nodes decreases significantly. The velocity of the nose decreases to a minimum of 784 m/s when the plastic strain reaches a maximum of 1.71. In stage I, the low-velocity regions correspond closely to the plastically deformed regions in the nose of the projectile.

**Figure 7:**
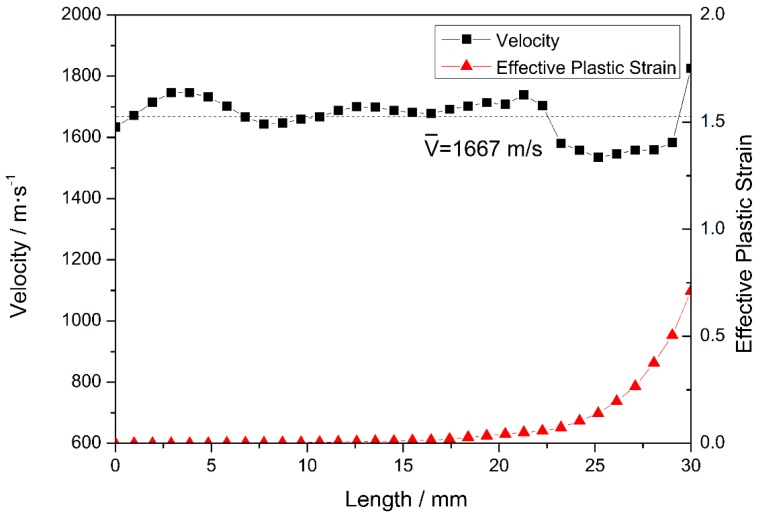
Node velocities and the corresponding element equivalent plastic strains at 12 μs.

The velocities of the nose and tail are similar and decrease simultaneously during stage II. However, the velocity of the nose fluctuates more than that of the tail, owing to the high-frequency interaction between the projectile and the target [34]; this interaction persists until the nose penetrates the steel plate at 18 μs. The result obtained at a typical time of t = 12 μs is further analyzed, as shown in Figure 7. At t = 12 μs, the length of the eroded projectile is 30 mm and the velocity of the nodes on the medial axis is ~1667 m/s. The first third of the projectile undergoes plastic deformation owing to the non-zero plastic strain in this region, which includes the nose element, where the maximum plastic strain is 0.71. The remaining two-thirds of the projectile undergo only elastic deformation. Interestingly, the node velocities of these two regions are basically the same, indicating that the node velocity is independent of the plastic deformation of the projectile. In contrast to the previous stage, the projectile reaches steady state in stage II.

**Figure 8:**
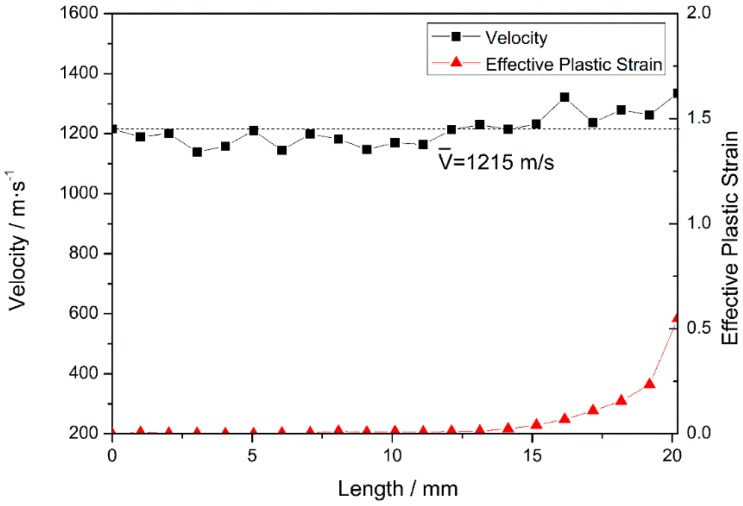
Node velocities and the corresponding element equivalent plastic strains at 40 μs.

In stage III, after penetrating the steel plate at 18 μs, the fragment starts to penetrate the PE laminate. The nose and tail decelerate in the same manner, and the fluctuation in the nose velocity decreases. T = 40 μs is selected as a typical time for further analysis. After erosion, the total length of the projectile is reduced to 20.2 mm (see Figure 8) and, as previously observed in stage II, the velocities of nodes on the medial axis fluctuate around 1215 m/s. When the fragment penetrates the target, the overall velocity of the projectile remains the same and fluctuates during the relaxation of stress waves, as reported in the literature [35]. The first third of the projectile undergoes plastic deformation at a certain plastic strain. In fact, the nose element experiences a maximum plastic strain of 0.55, and the other two-thirds of the projectile undergo only elastic deformation (as the plastic strain is zero). Compared with that occurring in stage II, the plastic deformation of the nose is less severe, because the strength of the PE laminate is lower than that of steel.

### 3.3. Analysis of the Internal Energy and Mass Loss of the Armor

**Figure 9:**
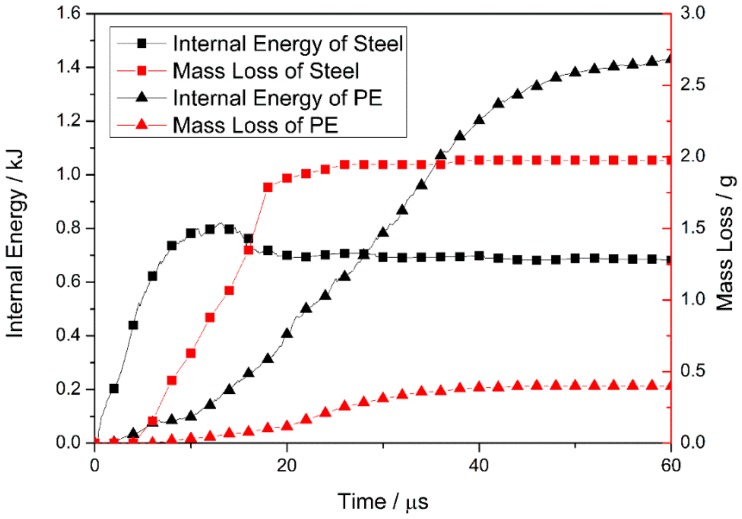
Internal energy and mass loss of the steel and pe laminate for times ranging from 0 to 60 μs.

The internal energy and mass loss of the steel and PE laminate are plotted for times ranging from 0 to 60 μs (see Figure 9), to determine the energy absorption of each layer during the projectile-target interactions.

At the beginning of penetration, the internal energy of the steel plate increases rapidly (owing to deformation), and is then released via element failure and deletion. For times ranging from 0 to 13 μs, energy accumulation outstrips energy release and, hence, the internal energy increases continuously. Element failure increases after 4 μs, and the rate of internal-energy increase is reduced. Subsequently, the internal energy starts to decrease, because the rate of energy accumulation in the plate is slower than the rate of release. After fragment penetration of the steel plate at 18 μs, the internal energy of the plate reaches a value of ~0.69 kJ, and the mass loss curve forms a plateau at 1.98 g. This indicates that the steel plate undergoes no further deformation or element failure.

The internal energy and mass loss curve indicates that the PE laminate undergoes deformation and element failure after energy absorption at T ~ 6 μs. This indicates that prior to complete fragment penetration of the steel plate, the laminate starts to absorb energy because it fits closely with the plate and deforms with the back bulge of the plate. Spalling of the plate, owing to the generation of a large reflected tension shock wave, may be prevented by the transmission of stress waves from the steel into the laminate and, therefore, the plate remains intact. The effective matching of two plates contributes to the ballistic performance of the composite armor. As the high-speed fragment penetrates the laminate, the mass loss of the laminate (owing to element failure) occurs mainly at T = 18–40 μs (see Figure 9), and the internal energy of the laminate increases significantly. The constant rates of mass loss and internal-energy increase of the laminate lead to a stable stage where the laminate absorbs energy via the continuous failure occurring during this period. In contrast, at T = 40–60 μs, the laminate experiences a constant mass loss of 0.40 g and the rate of internal-energy increase is reduced. This indicates that, in this period, the laminate absorbs the kinetic energy of the fragment mainly because of its ability to accommodate high levels of deformation.

When the fragment penetrates the steel plate, the internal energy of the plate increases from zero reaching a maximum of 0.82 kJ at 13 μs, decreases to 0.69 kJ at 18 μs, and remains approximately constant thereafter. The subsequent constant mass loss shows that the plate stops the absorption of kinetic energy. Before the projectile reaches the PE laminate, the internal energy of the laminate increases continuously, reaching a maximum value (1.43 kJ) at 60 μs. This value accounts for 68% of the total energy absorption of the target armor. Therefore, the PE laminate in the composite-structure armor plays an important role in the absorption of projectile kinetic energy during the penetration process.

## 4. Conclusions

The penetration of a 30CrMnMo-UHMWPE composite armor by a high-speed fragment is investigated with the aim of determining the ballistic performance thereof and revealing the underlying protective mechanism. Numerical simulation results of the penetration depth and residual mass of the fragment agree well with the experimental results. Further studies revealed that the nose of the projectile is in the non-equilibrium state at the initial stage of penetration, and the low-speed regions undergo plastic deformation. The subsequent nose-tail velocities of the projectile are almost identical and fluctuate together. Moreover, the results revealed that the effective combination of the steel plate and the PE laminate results in energy absorption by the PE, just before the projectile nose impacts the laminate. This early energy absorption plays a positive role in the ballistic performance of the composite armor. Further analysis indicates that the UHMWPE hot-pressed laminate absorbs energy via the continuous and stable failure of PE fibers during the initial stages of penetration. Subsequently, the laminate absorbs the kinetic energy of the projectile mainly through deformation until complete penetration occurs. The energy absorbed accounts for 68% of the energy consumed by the whole armor, indicating that the laminate plays a major role in the energy absorption during the simulated penetration process.

## Figures and Tables

**Table 1:** Material parameters in the FEM (finite element method) model.

**Johnson Cook Model**	**1045-Steel**	**30CrMnMo**	**Al**
ρ (g·cm^−3^)	7.85	7.85	2.78
E (Gpa)	210	200	69.0
G (Gpa)	80.8	75.9	27.0
PR	0.31	0.32	0.33
A (GPa)	0.507	1.18	0.554
B (GPa)	0.32	0.1625	0.351
C	0.28	0.058	0.009
n	0.064	0.28	0.37
m	1.06	1.15	1.09
D_1_	0.15	0.123	1.5
D_2_	0.72	0.0	0.0
D_3_	1.66	0.0	0.0
D_4_	0.005	0.694	0.0
D_5_	-0.84	0.501	0.0
**Orthotropic Elastic Model**		**UHMWPE**	
EA (GPa)	76.6	ρ (g·cm^−3^)	0.97
EB (GPa)	0.77	PRBA	0.013
EC (GPa)	76.6	PRCA	0.0
GAB (GPa)	2.0	PRCB	0.5
GBC (GPa)	2.0	-	-
GCA (GPa)	0.192	-	-

**Table 2:** Comparisons between experimental result and simulation.

Results	Experiment	Simulation	Relative Error
Penetration depth (mm)	14.0	15.3	9.3%
Residual mass (g)	6.2	6.6	6.5%
